# Dynamic modeling of folliculogenesis signaling pathways in the presence of miRNAs expression

**DOI:** 10.1186/s13048-017-0371-y

**Published:** 2017-12-19

**Authors:** Abolfazl Bahrami, Seyed Reza Miraie-Ashtiani, Mostafa Sadeghi, Ali Najafi, Reza Ranjbar

**Affiliations:** 1 Department of Animal Science, University college of Agriculture and Natural Resources, University of Tehran, Karaj, Iran; 2 Molecular Biology Research Center, Baqiyatallah University of Medical Sciences, Tehran, Iran

**Keywords:** Dynamic modeling, Folliculogenesis, TEK signaling, Ras/ERK/MYC, PI3K/AKT/mTORC1

## Abstract

**Background:**

TEK signaling plays a very important role in folliculogenesis. It activates Ras/ERK/MYC, PI3K/AKT/mTORC1 and ovarian steroidogenesis activation pathways. These are the main pathways for cell growth, differentiation, migration, adhesion, proliferation, survival and protein synthesis.

**Results:**

TEK signaling on each of the two important pathways where levels of pERK, pMYC, pAkt, pMCL1 and pEIF4EBP1 are increased in dominant follicles and pMYC is decreased in dominant follicles. Over activation of ERK and MYC which are the main cell growth and proliferation and over activation of Akt, MCl1, mTORC1 and EIF4EBP1 which are the main cell survival and protein synthesis factors act as promoting factors for folliculogenesis. In case of over expression of hsa-miR-30d-3p and hsa-miR-451a, MYC activity level is considerably increased in subordinate follicles. Our simulation results show that in the presence of has-miR-548v and bta-miR-22-3p, downstream factors of pathways are inhibited.

**Conclusions:**

Our work offers insight into the design of natural biological procedures and makes predictions that can guide further experimental studies on folliculogenesis pathways. Moreover, it defines a simple signal processing unit that may be useful for engineering synthetic biology and genes circuits to carry out cell-based computation.

**Electronic supplementary material:**

The online version of this article (doi:10.1186/s13048-017-0371-y) contains supplementary material, which is available to authorized users.

## Background

In the previous study [1], transcriptome profiling of granulosa cells from small (< 5 mm) and large (> 10 mm) follicles using high-throughput data sets were analyzed and “Integrated miRNA–mRNA Bipartite Network” was reconstructed. According to the next analysis, the most important signaling pathways involved in folliculogenesis were detected. These pathways were included Ras/ERK/MYC, PI3K/AKT/mTORC1 and ovarian steroidogenesis. Based on these findings, Ras/ERK/MYC and PI3K/AKT/mTORC1 pathways represent regulatory networks and fundamental signaling transduction for the majority of cellular physiological processes in folliculogenesis, such as growth, differentiation, migration, adhesion, proliferation, survival and protein synthesis. These pathways are mostly activated by alterations in ANGPT1, TEK, MYC, MAPK1, PIK3R1, MCL1 and EIF4EBP1 genes [1]. Activation of such pathways is responsible of uncontrolled cellular physiological processes and can contribute to drug discovery. Combination therapies with pharmacological inhibitors or activators of these follicles pathways may have potential uses for the control of folliculogenesis process. Comparison of between dominant and subordinate follicles is a good model to investigate the activation of Ras/ERK/MYC and the PI3K/AKT/mTORC1 pathways as it is frequently affected by miRNAs that causes the activation or inhibition of pathways [1]. Folliculogenesis is thought to occur by sequential accumulation of molecular alterations and genetic [2]. Although, the mechanisms underlying follicle development are still unknown, several genes and metabolic pathways have been shown to carry molecular alterations in folliculogenesis. Folliculogenesis exhibit mutations in the Ras/RAF/mitogen activated protein kinase (MAPK) pathway. This mutation is known to play a key role in cell growth and proliferation of follicles, through activation of the ERK pathway [1]. In particular, it occurs within the activation segment of the kinase domain and it results in an increased activity of the kinase itself. Constitutive activation of the kinase activity leads to unresponsitivity of negative feedback mechanisms within the ERK pathway [3]. Previous studies show that ERK and AKT pathways are activated in parallel and the evidence that PI3K/AKT and MAPK/ERK1/2 cascades are interconnected is described [4–6]. According to these findings, ERK and AKT pathways could represent targets for an otherwise devastating trait. Computational or dynamical modeling and Computer simulations are useful to analyze and to increase understanding of metabolic pathways [7], specially involved in folliculogenesis and their complex interactions. On the other hand, MYC activity must be controlled in response to different environmental cues. Previous studies have showed that MYC is regulated at multiple levels, including post-transcriptional regulation [8, 9] and autoregulation of MYC transcription [10]. More recent discoveries indicate that MYC is also regulated at the protein level by the Ras effector pathways [11–14]. The control of MYC dynamics by successive phosphorylation allows MYC to merge upstream signals from ERK and PI3K, which play key roles in controlling diverse cell fates [15, 16]. As well as, major advances in understanding cell survival and translation control have come from the discovery of the intimate relationship between growth factor, the mitogen and hormone-responsive phosphatidylinositol 3-kinase (PI3K) pathway and the energy-sensing target of rapamycin (TOR) pathway. The PI3K and TOR pathways converge on common downstream targets: the ribosomal S6 kinases (S6Ks) and the translation initiation factor 4E-binding proteins (4E-BPs). Since the discovery that the TOR inhibitor rapamycin is effective in treatment of cell caused by misregulation of players in the PI3K pathway, the importance of the interplay between these two pathways is gaining greater recognition [17]. In this work we survey here recent advances in our understanding of how the PI3K pathway impinges on the PI3K/AKT/mTORC1 pathway, how levels of expression in the PI3KR1 and EIF4EBP1pathways affect cell survival and translation control, and how the downstream targets of mTORC1 regulate cell survival. We develop a dynamic model that simulates both Ras/ERK/MYC and PI3K/AKT/mTORC1 pathways and their interactions for analyzing the reactions responsible for follicle development. Moreover, we modeled the behavior of the follicles in the presence of miRNAs expression, under the different stages of folliculogenesis.

There are several modeling approaches and their applications in current different research. These popular modeling approaches can simulate the dynamic changes of regulatory networks (metabolic pathways and signaling pathways), cell growth, and its environments, such as ordinary differential equations (ODEs), Petri nets, Boolean network, agent-based model, the system biology modeling approach considering genetic variation, and linear programming (LP) based model [18]. Of these, because of modeling base on ODEs is able to predict precisely and continuously manner of each node in the network during the time, so in our work, it was considered. Overall, this model may be used for an in silico lab to study the effects of potential activators or inhibitors that may improve the response to standard treatments.

## Methods

### Model description

**Fig. 1:**
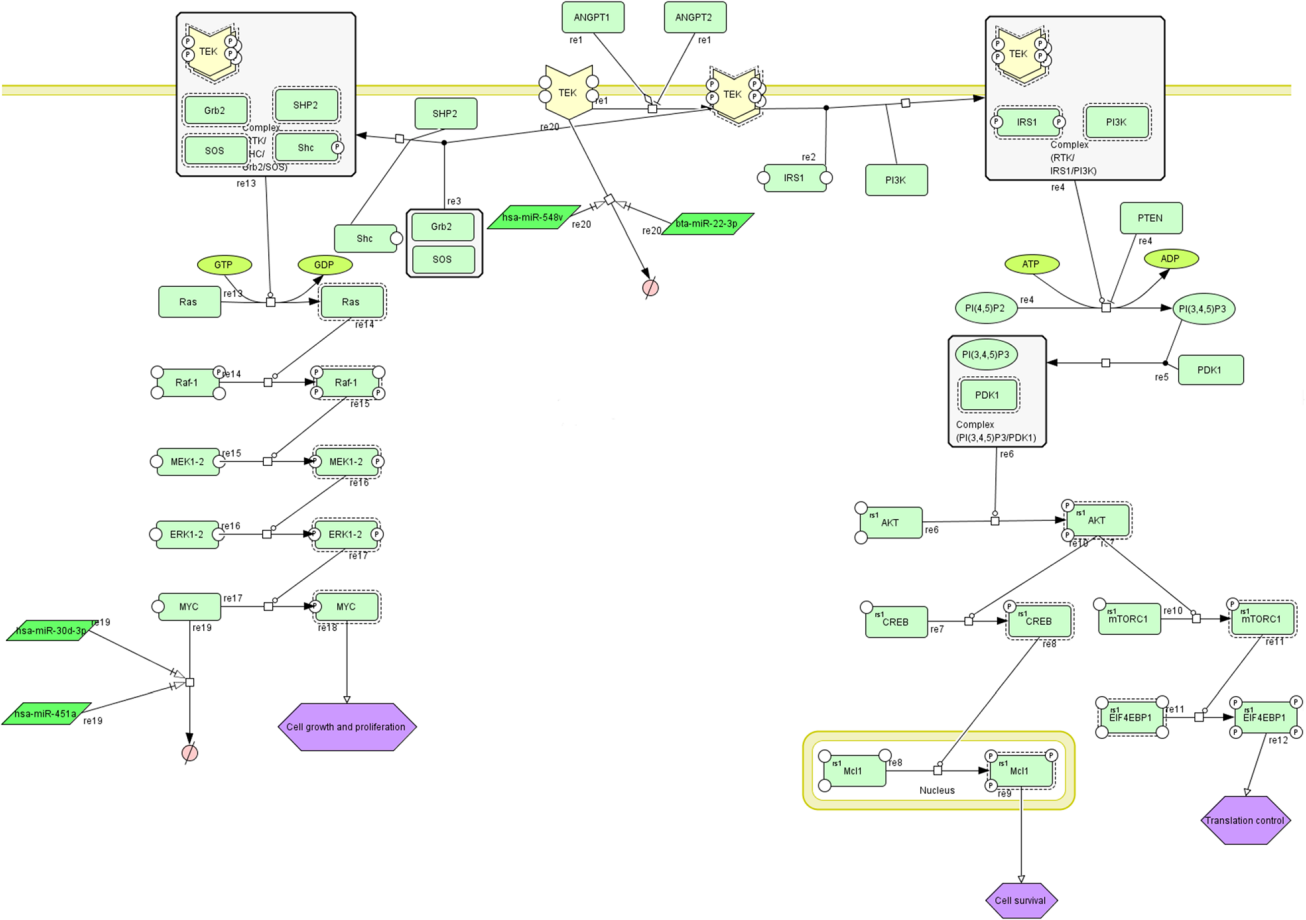
Schematic overview of the TEK signaling pathway which was built with CellDesigner 4.4

The related network for subordinate, dominant and dominant follicles in the presence of miRNAs were generated according to previous models and experimental observations that dealt with published signaling network dynamic behavior [19–38] (Additional file 1: Tables S1–S3). The network consists of two main pathways (Fig. 1) that play an important role in cell growth, proliferation, survival and protein synthesis. These pathways are activated by ANGPT1 and ANGPT2 attachment to TEK. After the interaction of ligand (ANGPT1 and ANGPT2 that these species were antagonist with each other) with the extracellular domain of TEK, the receptors undergo homodimerization that causes auto phosphorylation of certain tyrosine residues in the cytoplasmic end. Growth factor receptor-bound protein 2 (Grb2) binds phosphorylated tyrosines (pY1148, pY1086) and Src homology 2 domain containing transforming protein (Shc) binds pY1148 and pY1173 [39]. After C-terminal tail phosphorylation, the Shc adaptor binds its site and Grb2 attaches to Shc, and then SOS (GTP exchange protein for Ras) is employed by Grb2. In the next step SOS converts Ras-GDP into Ras-GTP which is the activated form of Ras [40, 41]. Activated Ras causes Raf phosphorylation and activation [42, 43]. There are three kinds of Raf in cells: Raf-1(C-Raf), A-Raf and B-Raf [44, 45]. Raf phosphorylates and activates the MEK (MAPK kinase). The activated MEK, phosphoralates and activates extracellular signal-regulated kinase 1 (ERK1) and ERK2. ERK1 and ERK2 phosphorylate MYC protein that leads to cell growth, proliferation and survival [1]. Two miRNAs are included hsa-miR-30d-3p and hsa-miR-451a suppress translation of MYC gene and result low concentration of MYC protein in dominant follicles. Phosphatidylinositol-3-kinase (PI3K) has two 85 and 110 KD subunits which are regulatory and catalytic subunits, respectively [46, 47]. After RTK phosphorylation, the regulatory subunit binds its phosphorylated Thyrosine site and then the catalytic and the regulatory subunits join together. Insulin receptor substrate-1(IRS1) phosphorylated. Under such conditions the, PI3K and IRS1 are activated and converts the membrane phosphatidyl inositol 4,5bisphosphate (PIP2) into phosphatidyl inositol 3,4,5-3phosphate (PIP3). PIP3 makes Akt activation in a way that PDK1 binds membrane PIP3, then PDK1, phosphorylates and activates Akt. Akt is activated and stimulates several factors such as phosphorylation of CREB and mTORC1. The CREB phosphorylates and activates Mcl1. On the other hand, mTORC1 phosphorylates and inactivates EIF4EBP1. So, Akt indirectly or directly regulates cell growth, survival and translation control through phosphorylating its substrates. Akt deactivation and the PI3K negative regulation are done by PTEN. PTEN is a phosphatase that removes phosphates group from the phosphatidylinositole 3, 4,5-3phosphate that results Akt inactivation [48]. Based on literature mining, TEK signaling network was modified for subordinate, dominant follicles and dominant follicles in the presence of miRNAs and reconstructed network were designed for the folliculogenesis state which is accessible in Additional file 1.

### Dynamical model

Systems biology involves network perspective of biological systems. The domain of systems biology could be from an individual reaction cycle of a cell, tissue, organ or even the whole organism. Computational modeling has an axial role in converting biological events to quantitative data for analyzing. For better understanding, such models should to reflect the systems as they exist because by only this way, reliable predictions are practical about them. One of the most usually practical methods in order to model biological systems is Ordinary Differential Equations (ODEs). A differential equation is explained as an equation showing the relationship between a function and some of the derivatives of that function. Fundamentally a differential equation determines how a variable such as [S], i.e. the concentration of S, alters over the time. This is done by inter relating the rate of change with the concentration at the moment [49–52]. Our suggested subordinate follicles (SF) and dominant follicles (DF) models are based on ODEs and involve 105 species, 112 reactions, 170 parameters and 1 rule. Our suggested dominant follilces in the presence of miRNAs (DF + miRNAs) model is based on ODEs and involves 110 species, 116 reactions, 178 parameters and 1 rule. In Additional file 1: Table S1, reactions are shown in SF and DF. The Additional file 1: Table S2 also show the initial values for non-zero species in SF, DF and DF + miRNAs respectively. On the other hand, in Additional file 1: Table S3, reactions are presented for DF + miRNAs model. The systems biology markup language (SBML) of our models is also presented in Additional file 2: SBML S1 (SF model), Additional file 3: SBML S2 (DF model) and Additional file 4: SBML S3 (DF + miRNAs model). SBML is a computer-readable format like XML for indicating models of biochemical reaction networks. For simulations, ODE15s routine from MATLAB 8.5 was used to solve ODEs. Here, a sample derivation regarding one of the ODEs (related to ANGPT1 and ANGPT2 and RTK binding) is reported; the reaction is considered as a second order one.

$$ ANGPT1+ TEK<\frac{K1}{Kr1}> ANGPT1- TEK $$

The reaction rate by which ANGPT1-RTK is produced is:

$$ v=K 1\left[ ANGPT 1\right]\left[ TEK\right]- Kr 1\left[ ANGPT 1- TEK\right] $$

Where K1 is the rate constant for the forward direction and Kr1 is for the reverse one.

## Results

### ANGPT1 and TEK over-expression in DF in the comparison of SF

**Fig. 2:**
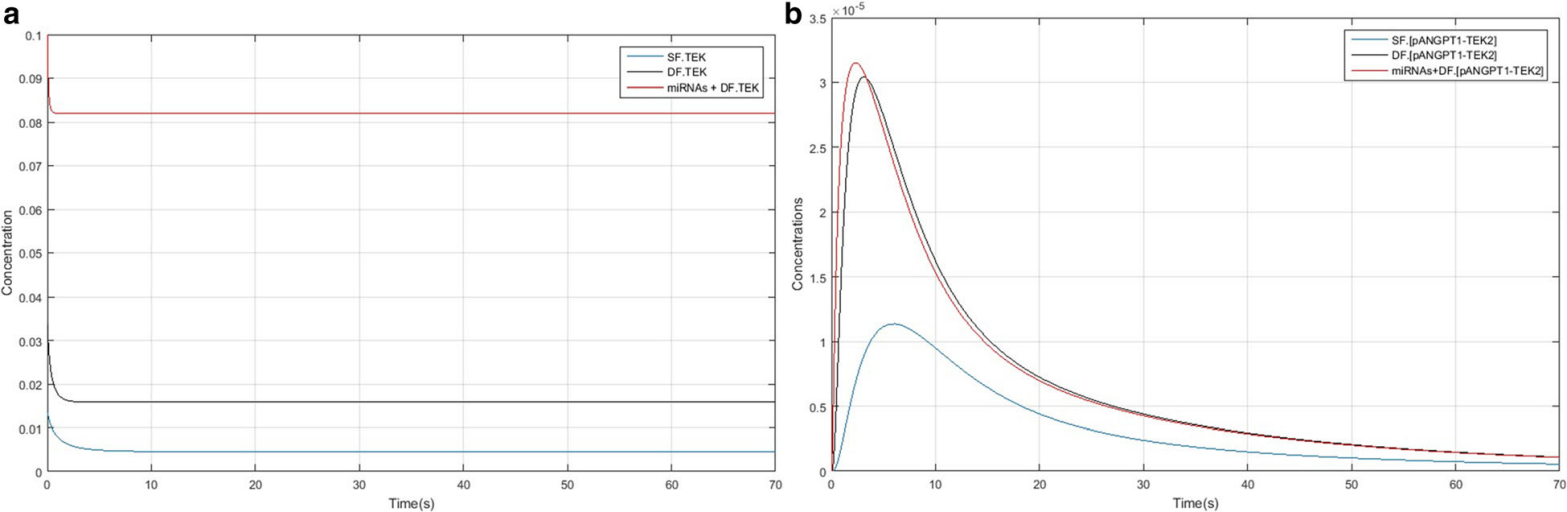
Computational simulation of TEK and ANGPT1-TEK autophosphorylation and internalization. **a** Kinetics of TEK concentration in different models in 70s. **b** Kinetics of ANGPT1-TEK phosphorylation and internalization in 70 s (blue, black and red line representing concentration in the subordinate follicles, dominant follicles and dominant follicles in the presence of miRNAs, respectively)

Figure 2a compared TEK concentration between SF (blue line), DF (black line) and DF + miRNAs (red line). In this figure TEK concentration in presence of miRNAs during the time was higher than SF and DF as well as, in DF higher than SF. On the other hand, Fig. 2b compared phosphorylated and activated ANGPT1-TEK is lagging relate to the SF in comparison of DF and DF + miRNAs but its concentration decreases with a mild slope. ANGPT1 was first detected as a major activator of TEK, resulting in a downstream activation of the phosphatidylinositol 3-kinase/Akt survival pathway, thereby promoting cell survival.

### Comparison of Ras/ERK/MYC pathways between SF, DF and DF + miRNAs model

**Fig. 3:**
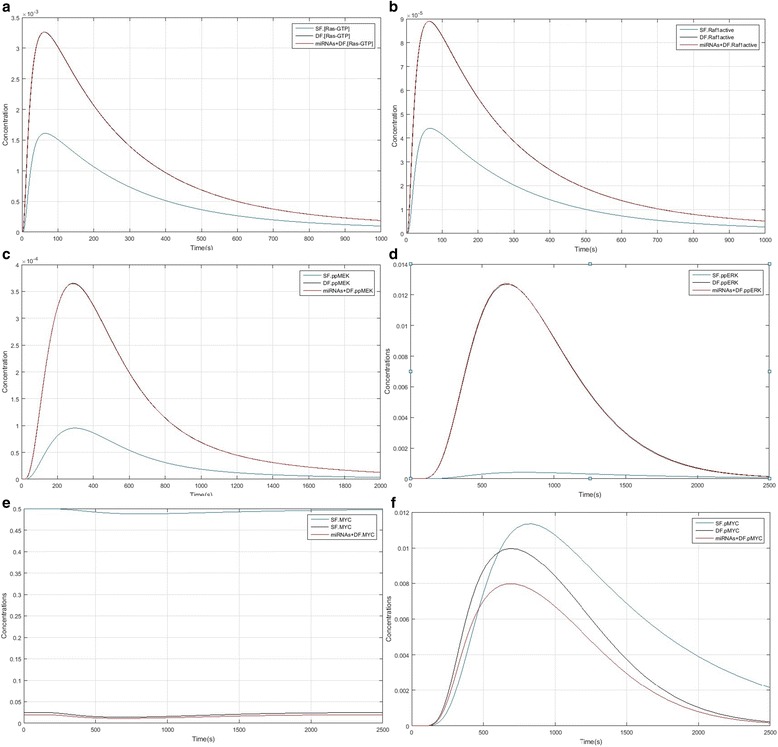
Important species kinetics comparison in Ras/ERK/MYC pathway between SF, DF and DF + miRNAs models. **a** Kinetics of Ras-GTP formation. **b** Kinetics of Raf1 activation. **c** Phosphorylation kinetics of MEK leading to ppMEK double phosphorylation. **d** Phosphorylation kinetics of ERK leading to ppERK double phosphorylation. **e** Concentration of MYC during the time. **f** Phosphorylation kinetics of MYC leading to ppMYC double phosphorylation: SF model factor (blue); DF model factors (black); DF + miRNAs model factors (red)

**Fig. 4:**
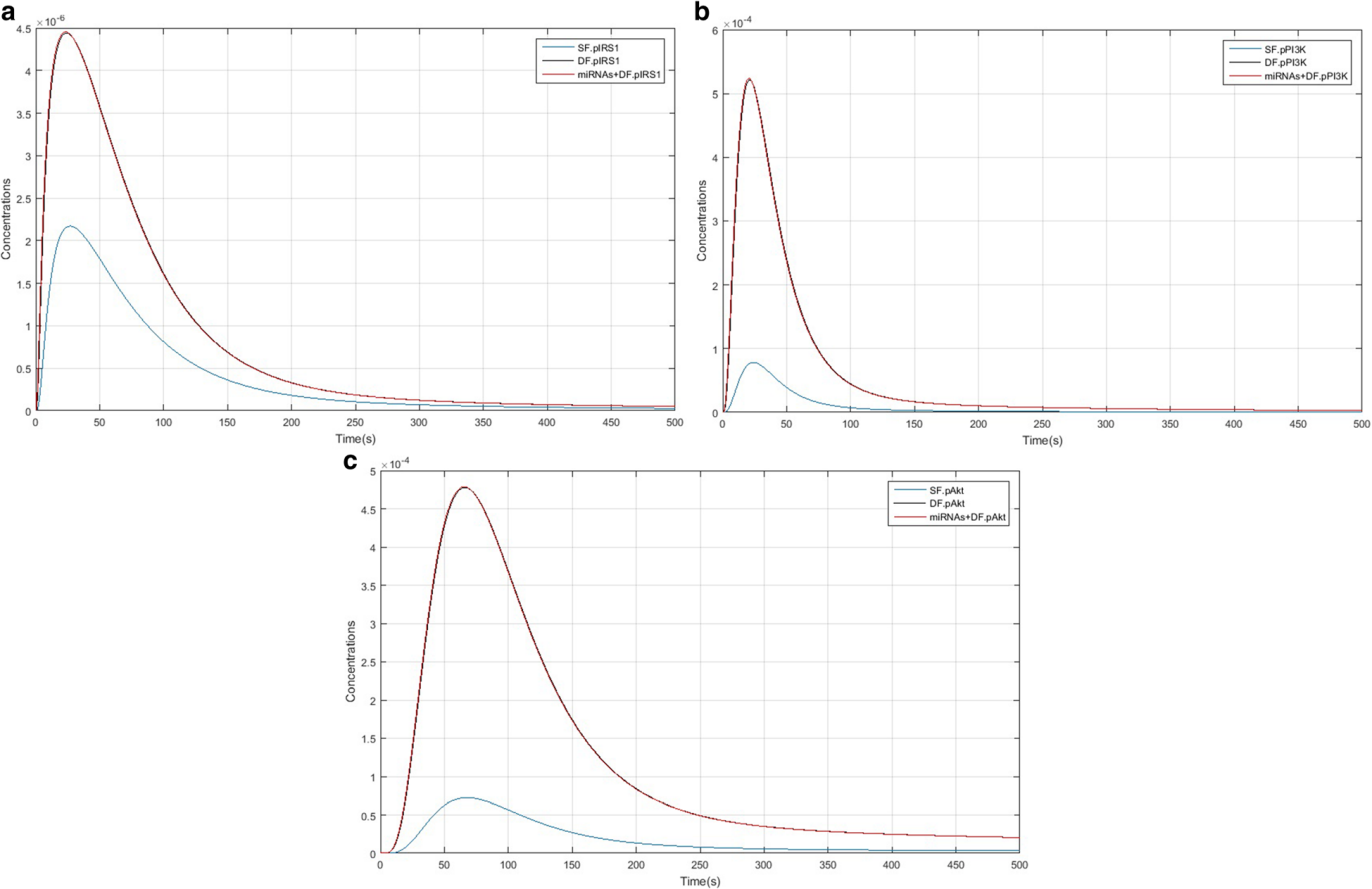
IRS1, PI3K and Akt species kinetics comparison in of PI3K/Akt/mTORC1 pathway between SF, DF and DF + miRNAs models. **a** Kinetics of IRS1 formation. **b** Kinetics of PI3K activation. **c** Phosphorylation kinetics of Akt leading to pAkt double phosphorylation: SF model factor (blue); DF model factors (black); DF + miRNAs model factors (red)

Our simulation results denoted that up-regulation of TEK, enhanced TEK and Ras expression in dominant follicles had a deep impact on Ras-ERK. Figure 4 compares important species kinetics in Ras/ERK/MYC between SF, DF and DF + miRNAs model and shows that Ras/ERK/MYC pathway activation kinetics is vastly different between the SF, DF and DF + miRNAs. Figure 3a compared Ras-GTP formation kinetics. Ras-GTP formation peak in SF is lagging relative to the DF and DF + miRNAs, but its concentration decreases with a mild slope. Of course lines relate to DF and DF + miRNAs were overlapping approximately and there isn’t different between them. Raf1 activation kinetics is also like to Ras-GTP formation (Fig. 3b). MEK downstream factor kinetics and ERK between the SF, DF and DF + miRNAs model are completely different. Figure 3c represents the MEK phosphorylation peak in DF and DF + miRNAs (red line overlapped black line) in about 280 s while in the SF model it is about 340 s. Phosphorylated MEK (ppMEK) in DF and DF + miRNAs are much higher than the SF model showing that in DF and DF + miRNAs MEK activity is higher and the active state period is longer for MEK. Figure 3d illustrates that phosphorylation or activity level of ERK (ppERK) in DF and DF + miRNAs (red and black lines) is not comparable to the SF in a way that conversely, SF model ppERK concentration is few and the simulation predicts that DF and DF + miRNAs ERK phosphorylation peak is nearly 650 s and for SF is about 950 s. As well as, the time period and the concentration of phosphorylated ERK in DF and DF + miRNAs is much more that the SF model. Figure 3e represent different concentration of MYC between SF, DF and DF + miRNAs. Concentration of MYC in subordinate follicles significantly is higher than both dominant follicles and dominant follicles in the presence of two miRNAs (hsa-miR-30d-3p and hsa-miR-451a) which both of them were up-regulated during the entire period. Also, the comparison between DF and DF + miRNAs model showed that concentration level of DF was higher than DF + miRNAs. Figure 3f show that phosphorylation or activity level of MYC (pMYC) in SF (blue line), DF (black line) and DF + miRNAs (red line) is considerable, SF model pMYC concentration is higher than DF and DF + miRNAs model and the simulation predicts that SF MYC phosphorylation peak is nearly 850 s and for DF and DF + miRNAs is about 700 s.

### Comparison of PI3K/AKT/mTORC1 pathways between SF, DF and DF + miRNAs model

**Fig. 5:**
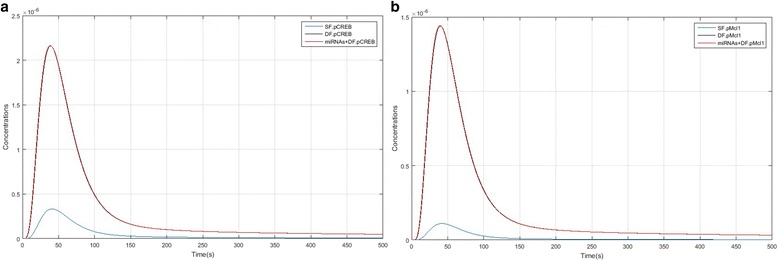
CREB and Mcl1 species kinetics comparison in of PI3K/Akt/mTORC1 pathway between SF, DF and DF + miRNAs models. **a** Kinetics of CREB formation. **b** Kinetics of Mcl1 activation: SF model factor (blue); DF model factors (black); DF + miRNAs model factors (red)

**Fig. 6:**
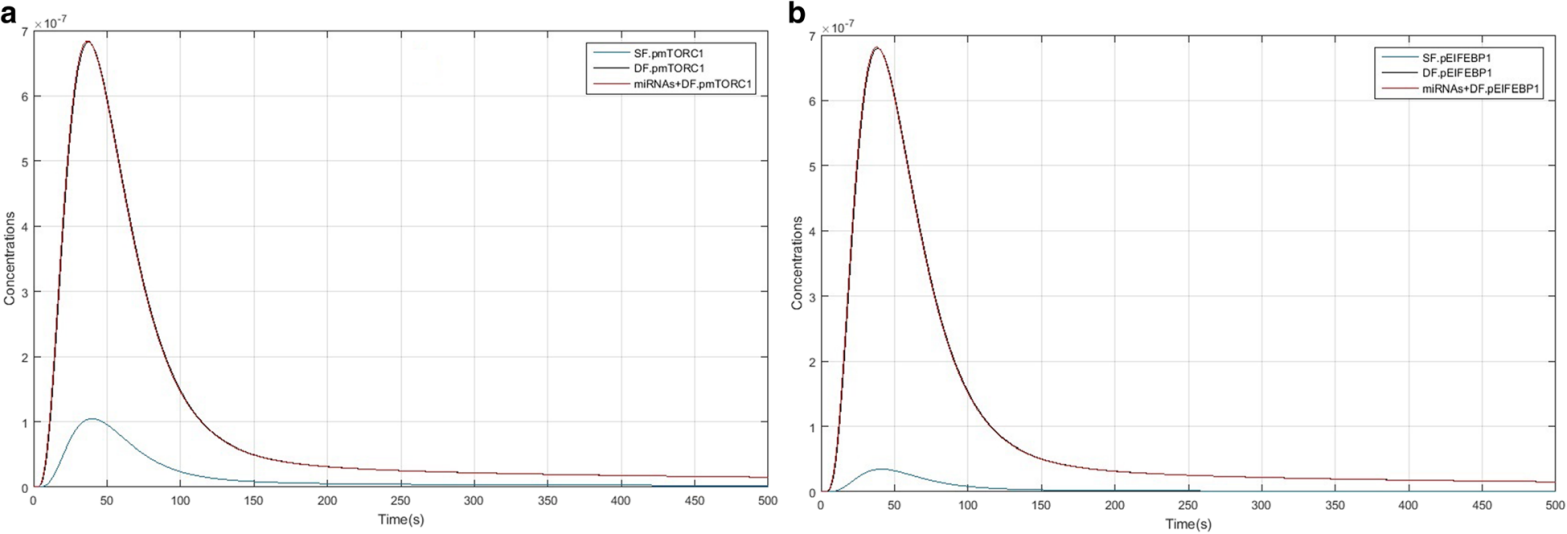
mTORC1 and EIFEBP1 species kinetics comparison in of PI3K/Akt/mTORC1 pathway between SF, DF and DF + miRNAs models. **a** Kinetics of pmTORC1 formation. **b** Kinetics of EIFEBP1 activation: SF model factor (blue); DF model factors (black); DF + miRNAs model factors (red)

Figure 4a, b and c compares IRS1, PI3K and Akt activation kinetics between SF, DF and the DF + miRNAs model. In Fig. 5a, b and c, it is showed that IRS1 (pIRS1), PI3K and Akt (pAkt) activity level in DF and DF + miRNAs (black and red line which lines were overlapping) are higher than the SF model. These models show that over-expression of ANGPT1 and TEK enhanced levels of these species, so high expression levels have a considerable effect on PI3K/Akt/mTORC1 pathway. Figure 5a and b show that formation of cytoplasmic CREB (pCREB) and nucleic Mcl1 (pMcl1) in DF and DF + miRNAs significantly have different kinetics compared to the SF model and the concentration level of pCREB and pMcl1 is much higher than the subordinate follicles. As well as, Fig. 6a and b compares mTORC1 and EIF4EBP1 activation kinetics between SF, DF and the DF + miRNAs model. pmTORC1 and pEIF4EBP1 formation peak in SF are lagging relative to the DF and DF + miRNAs. Of course lines relate to DF and DF + miRNAs were overlapping approximately and there isn’t different between them.

## Discussion

In the previous work, Bahrami et al. [1] analyzed the global transcriptome in both dominant follicles and subordinate follicles. Taking advantage of hierarchical cluster, they found the global gene expression is greatly altered, with 233 genes significantly upregulated and 44 genes significantly downregulated. In the KEGG pathway analysis of altered genes, they also found that Ras/ERK/MYC and PI3K/AKT/mTORC1 signaling pathways were highly enriched in deregulated genes. As well as ANGPT2 gene was downregulated. Besides the protein encoded by this gene is an antagonist of angiopoietin 1 (ANGPT1) and endothelial TEK tyrosine kinase [1]. Of the four identified angiopoietins (ANGPT1 to ANGPT4), the best characterized are ANGPT1 and ANGPT2. ANGPT1 and ANGPT2 have both been identified as ligands for TEK, a receptor expressed on endothelial cells, and they play important roles in angiogenesis, in concert [53–58]. There are a few studies about over-expression of TEK receptor in folliculogenesis process. In general, dynamics modeling of biological process in the reproduction events is rare and this study in the first report around computational modeling of ovary changes and biological procedures. In the previous study we reported over-expression of TEK receptor in dominant follicles. On the other hand, two miRNAs include has-miR-548v and bta-miR-22-3p which suppress RTK or TEK receptor, were down-regulated in dominant follicles. As well as, expression level of ANGPT1 and ANGPT2 were up-regulated and down-regulated, respectively [1]. ANGPT1binding to TEK maintains and stabilizes mature vessels by promoting interactions between endothelial cells and surrounding extra-cellular matrix. However, ANGPT2 shows context-dependent, antiangiogenic and proangiogenic activities. ANGPT2 was first identified as a natural antagonist for TEK that disrupts in vivo angiogenesis. Studies of ANGPT2 knockout animals reported that ANGPT2 is not need to embryonic vascular development, but is needed for postnatal, angiogenic remodeling [59]. ANGPT2 is only up-regulated at sites of active vascular remodeling, which involves vessel regression and destabilization [56]. There are many studies on ERK roles in biological process especially in cancer study. Activated ERK has been elicited Form 91% out of 101 head and neck cancer samples, 84% of 60 prostate cancer samples, 72% out of 90 breast cancer samples and 67% out of 74 gastric cancer samples respectively [60, 61]. Bahrami et al. [1] have revealed that ERK activation is related to RTK signaling in folliculogenesis. The MYC protein is a nuclear phosphoprotein that plays a role in cellular transformation, cell cycle progression, apoptosis, cell growth and proliferation [62]. Because of subordinate follicles critical need to cell growth and proliferation thus MYC protein concentration was high in these follicles. Bahrami et al. [1] disclosed the activity of PI3K/Akt/mTORC1 pathway in folliculogenesis. Mcl1 protein that relates to cell survival and cell adhesion has high concentration in the DF, because of these follicles need to survival for the ovulating process. On the other hand, this protein is an anti-apoptotic protein, which is a member of the Bcl-2 family, so involved in the regulation of apoptosis versus cell survival, and in the maintenance of viability but not of proliferation. EIF4EBP1 protein which relate to PI3K/AKT/mTORC1 signaling pathway and protein synthesis has high concentration in the DF. This protein is one member of a family of translation repressor proteins. The protein directly interacts with eukaryotic translation initiation factor 4E, which is a limiting component of the multisubunit complex that recruits 40S ribosomal subunits to the 5′ end of mRNAs. Interaction of this protein with eukaryotic translation initiation factor 4E inhibits complex assembly and represses translation. Since DF don’t need to additional growth, protein synthesis via high concentration of this protein was inhibited.

## Conclusion

In this study, for the first time we have made mathematical and dynamical models representing TEK signaling in subordinate and dominant follicles as well as dominant follicles in the presence of miRNAs and explore the different behaviors and dynamics of these models. For the first time we analyze the TEK signaling pathway specially involved in folliculogenesis. As well as, for the first time we have done dynamic modeling in the presence of differential expression of miRNAs. Since, TEK signaling is crucial for cell growth, survival, proliferation and translation control (protein synthesis); it might be the main reason for biological progression in folliculogenesis. TEK signaling activates Ras/ERK/MYC and PI3K/AKT/mTORC1 activation pathways. These two pathways are the main routes for cell growth, survival and proliferation [1]. Therefore over or down expression that lead to excessive activation of these pathways may cause folliculogenesis. This differential expression causes kinetic changes in downstream factors in the above pathways. This study has denoted how computational and dynamical models can be useful tools for exploring and comparing the biological behavior of signal transduction pathways as they can propose new hypotheses to define the observed biological data and help understand the dynamics of how the pathway functions. In addition, computational and dynamical models can be used to explore different trait states and propose how drug treatment could be improved to better combat the effects of the trait. We believe that our model is a good delegation of the PI3K/AKT/mTORC1 and Ras/ERK/MYC pathways. And we need to more investigation about dynamic behaviors involved in folliculogenesis in the future.

## Additional files

Additional file 1: **Tables S1 - S3.** (DOCX 125 kb)

Additional file 2: **SBML S1 (SF model).** (XML 158 kb)

Additional file 3: **SBML S2 (DF model).** (XML 158 kb)

Additional file 4: **SBML S3 (DF+miRNAs model).** (XML 163 kb)

## Abbreviations

ANGPT1: Angiopoietin-1
ANGPT2: Angiopoietin-2
DF: Dominant follicle
EIF4EBP1: Eukaryotic Translation Initiation Factor 4E Binding Protein 1
MAPK: Mitogen-Activated Protein Kinase
ODE: Ordinary differential equation
PI3K: Phosphoinositide 3-kinase
PIP2: Phosphatidyl inositol 4,5bisphosphate
SF: Subordinate follicle

## References

[1] Bahrami A, Miraie-Ashtiani SR, Sadeghi M, Najafi A (2017). miRNA-mRNA network involved in folliculogenesis interactome: systems biology approach. Reproduction.

[2] Rodgers RJ, Irving-Rodgers HF (2010). Morphological classification of bovine ovarian follicles. Reproduction.

[3] Ascierto PA, Kirkwood JM, Grob JJ, Simeone E, Grimaldi AM, Maio M (2012). The role of BRAF V600 mutation in melanoma. J Transl Med.

[4] Perkinton MS, Ip J, Wood GL, Crossthwaite AJ, Williams RJ (2002). Phosphatidylinositol 3-kinase is a central mediator of NMDA receptor signalling to MAP kinase (Erk1/2), Akt/PKB and CREB in striatal neurones. J Neurochem.

[5] York RD, Molliver DC, Grewal SS, Stenberg PE, McCleskey EW, Stork PJS (2000). Role of phosphoinositide 3-kinase and endocytosis in nerve growth factor-induced extracellular signal-regulated kinase activation via Ras and Rap1. Mol Cell Biol.

[6] Zhuang ZY, Xu H, Clapham DE, Ji RR (2004). Phosphatidylinositol 3-kinase activates ERK in primary sensory neurons and mediates inflammatory heat hyperalgesia through TRPV1 sensitization. J Neurosc.

[7] Gullo F, van der Garde M, Russo G, Pennisi M, Motta S, Pappalardo F (2015). Computational modeling of the expansion of human cord blood CD133+ hematopoietic stem/progenitor cells with different cytokine combinations. Bioinformatics.

[8] Endo T, Nadal-Ginard B (1986). Transcriptional and posttranscriptional control of c-myc during myogenesis: its mRNA remains inducible in differentiated cells and does not suppress the differentiated phenotype. Mol Cell Biol.

[9] Levine RA, McCormack JE, Buckler A, Sonenshein GE (1986). Transcriptional and posttranscriptional control of c-myc gene expression in WEHI 231 cells. Mol Cell Biol.

[10] Penn LJ, Brooks MW, Laufer EM, Land H (1990). Negative autoregulation of cmyc transcription. EMBO J.

[11] Sears R, Leone G, DeGregori J, Nevins JR (1999). Ras enhances Myc protein stability. Mol Cell.

[12] Sears R, Nuckolls F, Haura E, Taya Y, Tamai K (2000). Multiple Rasdependent phosphorylation pathways regulate Myc protein stability. Genes Dev.

[13] Yeh E, Cunningham M, Arnold H, Chasse D, Monteith T (2004). A signalling pathway controlling c-Myc degradation that impacts oncogenic transformation of human cells. Nat Cell Biol.

[14] Escamilla-Powers JR, Sears RC (2007). A conserved pathway that controls c-Myc protein stability through opposing phosphorylation events occurs in yeast. J Biol Chem.

[15] Vojtek AB, Der CJ (1998). Increasing complexity of the Ras signaling pathway. J Biol Chem.

[16] Ebisuya M, Kondoh K, Nishida E (2005). The duration, magnitude and compartmentalization of ERK MAP kinase activity: mechanisms for providing signaling specificity. J Cell Sci.

[17] Rameh LE, Cantley LC (1999). The role of phosphoinositide 3-kinase lipid products in cell function. J Biol Chem.

[18] Najafi A, Bidkhori G, Bozorgmehr JH (2014). Genome scale modeling in systems biology: algorithms and resources. Curr Genomics.

[19] Appuhamy JADRN, Hanigan MD (2011). Modeling the effects of insulin and amino acids on the phosphorylation of mTOR, Akt, and 4EBP1 in mammary cells. Modelling nutrient digestion and utilisation in farm animals.

[20] Dalle Pezze P, Nelson G, Otten EG, Korolchuk VI, Kirkwood TB, von Zglinicki T, Shanley DP (2014). Dynamic modelling of pathways to cellular senescence reveals strategies for targeted interventions. PLoS Comput Biol.

[21] Engin H, Üstünda Y, Tekin IO, Gökmen A, Ertop Ş, Ilikhan SU (2012). Plasma concentrations of angiopoietin-1, angiopoietin-2 and Tie-2 in colon cancer. Eur Cytokine Netw.

[22] Hsieh MY (2010). Spatio-temporal modeling of signaling protein recruitment to EGFR. BMC Syst Biol.

[23] Bidkhori G, Moeini A, Masoudi-Nejad A (2012). Modeling of tumor progression in NSCLC and intrinsic resistance to TKI in loss of PTEN expression. PLoS One.

[24] Kholodenko BN (1999). Quantification of short term signaling by the epidermal growth factor receptor. J Biol Chem.

[25] Kim SY, Herbst A, Tworkowski KA, Salghetti SE, Tansey WP (2003). Skp2 regulates Myc protein stability and activity. Mol Cell.

[26] Kiyatkin A (2006). Scaffolding protein Grb2-associated binder 1 sustains epidermal growth factor-induced mitogenic and survival signaling by multiple positive feedback loops. J Biol Chem.

[27] Lepique AP, Moraes MS, Rocha KM, Eichler CB, Hajj GNM (2004). c-Myc protein is stabilized by fibroblast growth factor 2 and destabilized by ACTH to control cell cycle in mouse Y1 adrenocortical cells. J Mol Endocrinol.

[28] Rabinowits G, Gerçel-Taylor C, Day JD, Taylor DD, Kloecker GH (2009). Exosomal MicroRNA: a diagnostic marker for lung cancer. Clin Lung Cancer.

[29] Salghetti SE, Kim SY, Tansey WP (1999). Destruction of Myc by ubiquitin-mediated proteolysis: cancer-associated and transforming mutations stabilize Myc. EMBO J.

[30] Sasagawa S (2005). Prediction and validation of the distinct dynamics of transient and sustained ERK activation. Nat Cell Biol.

[31] Sedaghat AR, Sherman A, Quon MJ (2002). Amathematicalmodelofmetabolic insulin signaling pathways. Am J Physiol Endocrinol Metab.

[32] Schoeberl B (2002). Computational modeling of the dynamics of the MAP kinase cascade activated by surface and internalized EGF receptors. Nat Biotechnol.

[33] Sonntag AG, Dalle Pezze P, Shanley DP, Thedieck K (2012). A modelling-experimental approach reveals insulin receptor substrate (IRS)-dependent regulation of adenosine monosphosphate-dependent kinase (AMPK) by insulin. FEBS J.

[34] Taylor DD, Taylor CG (2008). MicroRNA signatures of tumor-derived exosomes as diagnostic biomarkers of ovarian cancer. Gynecol Oncol.

[35] Ung CY (2008). Simulation of the regulation of EGFR endocytosis and EGFR-ERK signaling by endophilin-mediated RhoA-EGFR crosstalk. FEBS Lett.

[36] Yamada S, Taketomi T, Yoshimura A (2004). Model analysis of difference between EGF pathway and FGF pathway. Biochem Biophys Res Commun.

[37] Yamada S (2003). Control mechanism of JAK/STAT signal transduction pathway. FEBS Lett.

[38] Morozova N, Zinovyev A, Nonne N, Pritchard LL, Gorban AN, Harel-Bellan A (2012). Kinetic signatures of microRNA modes of action. RNA.

[39] Cetin Z, Ozbilim G, Erdogan A, Luleci G, Karauzum SB (2010). Evaluation of PTEN and Mcl-1 expressions in NSCLC expressing wild-type or mutated EGFR. Med Oncol.

[40] Li X, Huang Y, Jiang J, Frank SJ (2011). Synergy in ERK activation by cytokine receptors and tyrosine kinase growth factor receptors. Cell Signal.

[41] Martelli AM, Evangelisti C, Chiarini F, Grimaldi C, Cappellini A (2010). The emerging role of the phosphatidylinositol 3-kinase/Akt/mammalian target of rapamycin signaling network in normal myelopoiesis and leukemogenesis. Biochim Biophys Acta.

[42] Carpenter G, Cohen S (1990). Epidermal growth factor. J Biol Chem.

[43] Wiley HS, Shvartsman SY, Lauffenburger DA (2003). Computational modeling of the EGF-receptor system: a paradigm for systems biology. Trends Cell Biol.

[44] Marshall M (1995). Interactions between Ras and Raf: key regulatory proteins in cellular transformation. Mol Reprod Dev.

[45] Marais R, Light Y, Paterson HF, Marshall CJ (1995). Ras recruits Raf-1 to the plasma membrane for activation by tyrosine phosphorylation. EMBO J.

[46] Zhao L, Vogt PK (2010). Hot-spot mutations in p110alpha of phosphatidylinositol 3-kinase (pI3K): differential interactions with the regulatory subunit p85 and with RAS. Cell Cycle.

[47] Franke TF, Kaplan DR, Cantley LC, Toker A (1997). Direct regulation of the Akt proto-oncogene product by phosphatidylinositol-3,4-bisphosphate. Science.

[48] Silva A, Yunes JA, Cardoso BA, Martins LR, Jotta PY (2008). PTEN posttranslational inactivation and hyperactivation of the PI3K/Akt pathway sustain primary T cell leukemia viability. J Clin Invest.

[49] Kitano H (2002). Systems biology: a brief overview. Science.

[50] Suresh Babu CV, Joo Song E, Yoo YS (2006). Modeling and simulation in signal transduction pathways: a systems biology approach. Biochimie.

[51] Kirschner MW (2005). The meaning of systems biology. Cell.

[52] Orton RJ, Sturm OE, Vyshemirsky V, Calder M, Gilbert DR (2005). Computational modelling of the receptor-tyrosine-kinase-activated MAPK pathway. Biochem J.

[53] Fox SB, Gatter KC, Harris AL (1996). Tumor angiogenesis. J Pathol.

[54] Davis S, Aldrich TH, Jones PF (1996). Isolation of Ang-1, a ligand for the Tie2 receptor, by secretion-trap expression cloning. Cell.

[55] Suri C, Jones PF, Patan S (1996). Requisite role of Ang-1, a ligand for the Tie2 receptor, during embryonic angiogenesis. Cell.

[56] Maisonpierre PC, Suri C, Jones PF (1997). Angiopoietin-2, a natural antagonist for Tie2 that disrupts in vivo angiogenesis. Science.

[57] Runting AS, Stacker SA, Wilks AF (1993). Tie2, a putative protein tyrosine kinase from a new class of cell surface receptor. Growth Factors.

[58] Hicklin DJ, Ellis LM (2005). Role of the vascular endothelial growth factor pathway in tumor growth and angiogenesis. J Clin Oncol.

[59] Gale NW, Thurston G, Hackett SF (2002). Angiopoietin-2 is required for postnatal angiogenesis and lymphatic patterning, and only the latter role is rescued by angiopoietin-1. Dev Cell.

[60] Gioeli D, Mandell JW, Petroni GR, Frierson HF, Weber MJ (1999). Activation of mitogen-activated protein kinase associated with prostate cancer progression. Cancer Res.

[61] Gee JM, Robertson JF, Ellis IO, Nicholson RI (2001). Phosphorylation of ERK1/2 mitogen-activated protein kinase is associated with poor response to anti-hormonal therapy and decreased patient survival in clinical breast cancer. Int J Cancer.

[62] Camarda R, Zhou AY, Kohnz RA, Balakrishnan S, Mahieu C, Anderton B, Eyob H, Kajimura S, Tward A, Krings G, Nomura DK, Goga A (2016). Inhibition of fatty acid oxidation as a therapy for MYC-overexpressing triple-negative breast cancer. Nat Med.

