# Supplementary material for: Dynamic modeling of folliculogenesis signaling pathways in the presence of miRNAs expression
Source: J Ovarian Res. 2017 Dec 19;10:76. doi: 10.1186/s13048-017-0371-y (PMC5735818; doi:10.1186/s13048-017-0371-y)
Supplement: Supplementary file 1 — Tables S1 - S3. (DOCX 125 kb) [file 13048_2017_371_MOESM1_ESM.docx]

**Additional file 1**

**Table S1**

1. **Subordinate follilces (SF) and dominant follicles (DF) reactions**

Note: Michaelis Menten constants are given in µM, first order rate constants in s^-1^ and second order rate constants in µM^-1^ s^-1^

| **reactions** | | **parameters** | | **References** |
| --- | --- | --- | --- | --- |
| **r1** | **ANGPT1 + TEK <-> [ANGPT1-TEK]** | **K1= 100** | **Kr1= 0.0038** | (**Engin et al., 2012)** |
| **r2** | **[ANGPT1-TEK] + [ANGPT1-TEK] <-> [ANGPT1-TEK2]** | **K2= 10** | **Kr2= 0.02** | **(Engin et al., 2012)** |
| **r3** | **[ANGPT1-TEK2] -> [pANGPT1-TEK2]** | **K3=2.014** |  | **(Engin et al., 2012)** |
| **r4** | **[pANGPT1-TEK2] + SHP2 <-> [pANGPT1-TEK2-SHP2]** | **K4=3.114** | **Kr4=0.2** | **(**[**Yamada et al, 2004**](#_ENREF_102)**)** |
| **r5** | **[pANGPT1-TEK2-SHP2] -> [ANGPT1-TEK2] + SHP2** | **K5=2.661** |  | **(Yamada et al, 2004)** |
| **r6** | **[pANGPT1-TEK2] + Shc <-> [pANGPT1-TEK2-Shc]** | **K6=90** | **Kr6=0.6** | **(**[**Yamada et al, 2004**](#_ENREF_102)**; Estimation)** |
| **r7** | **[pANGPT1-TEK2-Shc] -> [pANGPT1-TEK2-pShc]** | **K7=0.5838** |  | **(Yamada et al, 2004; Estimation)** |
| **r8** | **[pANGPT1-TEK2-pShc] <-> [pANGPT1-TEK2] + pShc** | **K8=4.481** | **Kr8=0.3** | **(Yamada et al, 2004; Estimation)** |
| **r9** | **pShc + SHP <-> [pShc-SHP2]** | **K9=3.114** | **Kr9=0.2** | **(**[**Yamada et al, 2004**](#_ENREF_102)**)** |
| **r10** | **[pShc-SHP2] -> Shc + SHP2** | **K10=0.2661** |  | **(**[**Yamada et al, 2004**](#_ENREF_102)**)** |
| **r11** | **pShc -> Shc** | **K11=0.005** |  | **(Yamada et al, 2004; Estimation)** |
| **r12** | **[pANGPT1-TEK2-pShc] + Grb2 <-> [pANGPT1-TEK2-pShc-Grb2]** | **K12=3** | **Kr12=0.1** | **(**[**Kholodenko et al, 1999**](#_ENREF_38)**)** |
| **r13** | **[pANGPT1-TEK2-pShc-Grb2] + SHP2 <-> [pANGPT1-TEK2-pShc-Grb2-SHP2]** | **K13=10** | **Kr13=1** | **(**[**Yamada et al, 2004**](#_ENREF_102)**)** |
| **r14** | **[pANGPT1-TEK2-pShc-Grb2-SHP2] -> [ANGPT1-TEK2] + pShc + Grb2 + SHP2** | **K14=2.661** |  | **(**[**Yamada et al, 2004**](#_ENREF_102)**)** |
| **r15** | **[pANGPT1-TEK2-pShc-Grb2] + SOS <-> [pANGPT1-TEK2-pShc-Grb2-SOS]** | **K15=10** | **Kr15=0.0214** | **(**[**Kholodenko et al, 1999**](#_ENREF_38)**)** |
| **r16** | **Grb2 + SOS <-> [Grb2-SOS]** | **K16=0.1** | **Kr16=0.0015** | **(**[**Kholodenko et al, 1999**](#_ENREF_38)**)** |
| **r17** | **[pANGPT1-TEK2-pShc] + [Grb2-SOS] <-> [pANGPT1-TEK2-pShc-Grb2-SOS]** | **K17=10** | **Kr17=0.045** | **(**[**Kholodenko et al, 1999**](#_ENREF_38)**)** |
| **r18** | **[pANGPT1-TEK2] + Grb2 <-> [pANGPT1-TEK2-Grb2]** | **K18=3** | **Kr18=0.05** | **(**[**Kholodenko et al, 1999**](#_ENREF_38)**)** |
| **r19** | **[pANGPT1-TEK2-Grb2] + SHP2 <-> [pANGPT1-TEK2-Grb2-SHP2]** | **K19=10** | **Kr19=1** | **(**[**Yamada et al, 2004**](#_ENREF_102)**)** |
| **r20** | **[pANGPT1-TEK2-Grb2-SHP2] -> [ANGPT1-TEK2] + Grb2 + SHP2** | **K20=2.661** |  | **(**[**Yamada et al, 2004**](#_ENREF_102)**)** |
| **r21** | **[pANGPT1-TEK2-Grb2] + SOS <-> [pANGPT1-TEK2-Grb2-SOS]** | **K21=10** | **Kr21=0.06** | **(**[**Kholodenko et al, 1999**](#_ENREF_38)**)** |
| **r22** | **[pANGPT1-TEK2] + [Grb2-SOS] <-> [pANGPT1-TEK2-Grb2-SOS]** | **K22=2.734** | **Kr22=0.025** | **(**[**Yamada et al, 2004**](#_ENREF_102)**)** |
| **r23** | **[pANGPT1-TEK2-pShc-Grb2-SOS] + [Ras-GDP] <-> [pANGPT1-TEK2-pShc-Grb2-SOS-Ras-GDP]** | **K23=202.9** | **Kr23=0.18** | **(Yamada et al, 2004; Estimation)** |
| **r24** | **[pANGPT1-TEK2-pShc-Grb2-SOS-Ras-GDP] -> [pANGPT1-TEK2-pShc-Grb2-SOS] + [Ras-GTP]** | **K24=0.1434** |  | **(Yamada et al, 2004; Estimation)** |
| **r25** | **[pANGPT1-TEK2-Grb2-SOS] + [Ras-GDP] <-> [pANGPT1-TEK2-Grb2-SOS-Ras-GDP]** | **K25=202.9** | **Kr25=0.18** | **(Yamada et al, 2004; Estimation)** |
| **r26** | **[pANGPT1-TEK2-Grb2-SOS-Ras-GDP] -> [pANGPT1-TEK2-Grb2-SOS] + [Ras-GTP]** | **K26=0.1434** |  | **(Yamada et al, 2004; Estimation)** |
| **r27** | **[pANGPT1-TEK2-Grb2-SOS] + [Ras-GDP] <-> [pEGF-EGFR2-Grb2-SOS-Ras-GDP]** | **K27=202.9** | **Kr27=0.18** | **(**[**Yamada et al, 2004**](#_ENREF_102)**)** |
| **r28** | **[Ras-GTP] -> [Ras-GDP]** | **K28=0.000167** |  | **(Yamada et al, 2004; Estimation)** |
| **r29** | **[pANGPT1-TEK2-pShc-Grb2-SOS-Ras-GDP] -> [pANGPT1-TEK2-pShc-Grb2-SOS] + [Ras-GTP]** | **K29=0.1434** |  | **(Yamada et al, 2004; Estimation)** |
| **r30** | **[Ras-GTP] + [Ras-GAP] <-> [Ras-GTP-Ras-GAP]** | **K30=2.854** | **Kr30=0.96** | **(**[**Yamada et al, 2004**](#_ENREF_102)**)** |
| **r31** | **[Ras-GTP-Ras-GAP] -> [Ras-GDP] + [Ras-GAP]** | **K31=7.76** |  | **(**[**Yamada et al, 2004**](#_ENREF_102)**)** |
| **r32** | **[pANGPT1-TEK2] + [Ras-GAP] <-> [pANGPT1-TEK2-Ras-GAP]** | **K32=0.1** | **Kr32=0.01** | **(**[**Yamada et al, 2004**](#_ENREF_102)**)** |
| **r33** | **[pANGPT1-TEK2-Ras-GAP] + [Ras-GTP] <-> [pANGPT1-TEK2-Ras-GAP-Ras-GTP]** | **K33=2.845** | **Kr33=0.96** | **(**[**Yamada et al, 2004**](#_ENREF_102)**)** |
| **r34** | **[pANGPT1-TEK2-Ras-GAP-Ras-GTP] -> [pANGPT1-TEK2-Ras-GAP] + [Ras-GDP]** | **K34=7.76** |  | **(**[**Yamada et al, 2004**](#_ENREF_102)**)** |
| **r35** | **[pANGPT1-TEK2-Ras-GAP] + SHP2 <-> [pANGPT1-TEK2-Ras-GAP-SHP2]** | **K35=3.114** | **Kr35=0.2** | **(**[**Yamada et al, 2004**](#_ENREF_102)**)** |
| **r36** | **[pANGPT1-TEK2-Ras-GAP-SHP2] -> [ANGPT1-TEK2] + [Ras-GAP] + SHP2** | **K36=2.661** |  | **(**[**Yamada et al, 2004**](#_ENREF_102)**)** |
| **r37** | **Raf1 + [Ras-GTP] <-> [Raf1-Ras-GTP]** | **K37=1.75** | **Kr37=0.05** | **(Yamada et al, 2004; Estimation)** |
| **r38** | **[Raf1-Ras-GTP] -> Raf1active + [Ras-GTP]** | **K38=0.7624** |  | **(Yamada et al, 2004; Estimation)** |
| **r39** | **Raf1active + MEK <-> [Raf1active-MEK]** | **K39=4** | **Kr39=0.01833** | **Bidkhori etal, 2012;** [**Schoeberl et al, 2002**](#_ENREF_80)**;** [**Ung et al, 2008**](#_ENREF_92)**)** |
| **r40** | **[Raf1active-MEK] -> Raf1active + pMEK** | **K40=3.5** |  | **Bidkhori etal, 2012;** [**Schoeberl et al, 2002**](#_ENREF_80)**;** [**Ung et al, 2008**](#_ENREF_92)**)** |
| **r41** | **Raf1active + pMEK <-> [Raf1active-pMEK]** | **K41=4** | **Kr41=0.01833** | **Bidkhori etal, 2012;** [**Schoeberl et al, 2002**](#_ENREF_80)**;** [**Ung et al, 2008**](#_ENREF_92)**)** |
| **r42** | **[Raf1active-pMEK] -> Raf1active + ppMEK** | **K42=2.9** |  | **Bidkhori etal, 2012;** [**Schoeberl et al, 2002**](#_ENREF_80)**;** [**Ung et al, 2008**](#_ENREF_92)**)** |
| **r43** | **ppMEK + ERK <-> [ppMEK-ERK]** | **K43=3** | **Kr43=0.033** | **Bidkhori etal, 2012;** [**Schoeberl et al, 2002**](#_ENREF_80)**;** [**Ung et al, 2008**](#_ENREF_92)**)** |
| **r44** | **[ppMEK-ERK] -> ppMEK + pERK** | **K44=16** |  | **Bidkhori etal, 2012;** [**Schoeberl et al, 2002**](#_ENREF_80)**;** [**Ung et al, 2008**](#_ENREF_92)**)** |
| **r45** | **ppMEK + pERK <-> [ppMEK-pERK]** | **K45=3** | **Kr45=0.033** | **Bidkhori etal, 2012;** [**Schoeberl et al, 2002**](#_ENREF_80)**;** [**Ung et al, 2008**](#_ENREF_92)**)** |
| **r46** | **[ppMEK-pERK] -> ppMEK + ppERK** | **K46=5.7** |  | **Bidkhori etal, 2012;** [**Schoeberl et al, 2002**](#_ENREF_80)**;** [**Ung et al, 2008**](#_ENREF_92)**)** |
| **r47** | **ppERK + MYC <-> [ppERK-MYC]** | **K47=2** | **Kr47=0.022** | **Bidkhori etal, 2012;** [**Schoeberl et al, 2002**](#_ENREF_80)**;** [**Ung et al, 2008**](#_ENREF_92)**)** |
| **r48** | **[ppERK-MYC]-> ppERK + pMYC** | **K48= 0.038** |  | **Bidkhori etal, 2012;** [**Schoeberl et al, 2002**](#_ENREF_80)**;** [**Ung et al, 2008**](#_ENREF_92)**)** |
| **r49** | **pMYC -> MYC** | **K49=0.034** |  | **Bidkhori etal, 2012;** [**Schoeberl et al, 2002**](#_ENREF_80)**;** [**Ung et al, 2008**](#_ENREF_92)**)** |
| **r50** | **Raf1active + Pase <-> [Raf1active-Pase]** | **K50=71.7** | **Kr50=0.2** | **Bidkhori etal, 2012;** [**Schoeberl et al, 2002**](#_ENREF_80)**;** [**Ung et al, 2008**](#_ENREF_92)**)** |
| **r51** | **[Raf1active-Pase] -> Raf1 + Pase** | **K51=1** |  | **Bidkhori etal, 2012;** [**Schoeberl et al, 2002**](#_ENREF_80)**;** [**Ung et al, 2008**](#_ENREF_92)**)** |
| **r52** | **ppMEK + Pase2 <-> [ppMEK-Pase2]** | **K52=14.3** | **Kr52=0.8** | **Bidkhori etal, 2012;** [**Schoeberl et al, 2002**](#_ENREF_80)**;** [**Ung et al, 2008**](#_ENREF_92)**)** |
| **r53** | **[ppMEK-Pase2] -> pMEK + Pase2** | **K53=0.058** |  | **Bidkhori etal, 2012;** [**Schoeberl et al, 2002**](#_ENREF_80)**;** [**Ung et al, 2008**](#_ENREF_92)**)** |
| **r54** | **pMEK + Pase2 <-> [pMEK-Pase2]** | **K54=0.25** | **Kr54=0.5** | **Bidkhori etal, 2012;** [**Schoeberl et al, 2002**](#_ENREF_80)**;** [**Ung et al, 2008**](#_ENREF_92)**)** |
| **r55** | **[pMEK-Pase2] -> MEK + Pase2** | **K55=0.58** |  | **Bidkhori etal, 2012;** [**Schoeberl et al, 2002**](#_ENREF_80)**;** [**Ung et al, 2008**](#_ENREF_92)**)** |
| **r56** | **ppERK + Pase3 <-> [ppERK-Pase3]** | **K56=7** | **Kr56=0.6** | **Bidkhori etal, 2012;** [**Schoeberl et al, 2002**](#_ENREF_80)**;** [**Ung et al, 2008**](#_ENREF_92)**)** |
| **r57** | **[ppERK-Pase3] -> pERK + Pase3** | **K57=0.27** |  | **Bidkhori etal, 2012;** [**Schoeberl et al, 2002**](#_ENREF_80)**;** [**Ung et al, 2008**](#_ENREF_92)**)** |
| **r58** | **pERK + Pase3 <-> [pERK-Pase3]** | **K58=5** | **Kr58=0.5** | **Bidkhori etal, 2012;** [**Schoeberl et al, 2002**](#_ENREF_80)**;** [**Ung et al, 2008**](#_ENREF_92)**)** |
| **r59** | **[pERK-Pase3] -> ERK + Pase3** | **K59=0.3** |  | **Bidkhori etal, 2012;** [**Schoeberl et al, 2002**](#_ENREF_80)**;** [**Ung et al, 2008**](#_ENREF_92)**)** |
| **r60** | **ppERK + [pANGPT1-TEK2-pShc-Grb2-SOS] <-> [ppERK-pANGPT1-TEK2-pShc-Grb2-SOS]** | **K60=8.898** | **Kr60=1** | **(**[**Yamada et al, 2004**](#_ENREF_102)**)** |
| **r61** | **[ppERK-pANGPT1-TEK2-pShc-Grb2-SOS] -> ppERK + [pANGPT1-TEK2] + pShc + Grb2 + pSOS** | **K61=0.0426** |  | **(**[**Yamada et al, 2004**](#_ENREF_102)**)** |
| **r62** | **ppERK + [pANGPT1-TEK2-Grb2-SOS] <-> [ppERK-pANGPT1-TEK2-Grb2-SOS]** | **K62=8.898** | **Kr62=1** | **(**[**Yamada et al, 2004**](#_ENREF_102)**)** |
| **r63** | **[ppERK-pANGPT1-TEK2-Grb2-SOS] -> ppERK + [pANGPT1-TEK2] + Grb2 + pSOS** | **K63=0.0426** |  | **(**[**Yamada et al, 2004**](#_ENREF_102)**)** |
| **r64** | **pSOS -> SOS** | **K64=0.002** |  | **(**[**Sasagawa et al, 2005**](#_ENREF_79)**)** |
| **r65** | **[pANGPT1-TEK2-pShc-Grb2-SOS] + cbl <-> [pANGPT1-TEK2-pShc-Grb2-SOS-cbl]** | **K65=0.5** | **Kr65=0.005** | **(**[**Yamada et al, 2004**](#_ENREF_102)**)** |
| **r66** | **[pANGPT1-TEK2-pShc-Grb2-SOS-cbl] + EPn <-> [pANGPT1-TEK2-pShc-Grb2-SOS-cbl-EPn]** | **K66=5** | **Kr66=0.1** | **(**[**Yamada et al, 2004**](#_ENREF_102)**)** |
| **r67** | **[pANGPT1-TEK2-pShc-Grb2-SOS-cbl-EPn] -> cbl + [Grb2-SOS] + EPn + pShc** | **K67=0.001** |  | **(**[**Yamada et al, 2004**](#_ENREF_102)**)** |
| **r68** | **[pANGPT1-TEK2-Grb2-SOS] + cbl <-> [pANGPT1-TEK2-Grb2-SOS-cbl]** | **K68=0.5** | **Kr68=0.005** | **(**[**Yamada et al, 2004**](#_ENREF_102)**)** |
| **r69** | **[pANGPT1-TEK2-Grb2-SOS-cbl] + EPn <-> [pANGPT1-TEK2-Grb2-SOS-cbl-EPn]** | **K69=5** | **Kr69=0.1** | **(**[**Yamada et al, 2004**](#_ENREF_102)**)** |
| **r70** | **[pANGPT1-TEK2-Grb2-SOS-cbl-EPn] -> cbl + [Grb2-SOS] + EPn** | **K70=0.001** |  | **(**[**Yamada et al, 2004**](#_ENREF_102)**)** |
| **r71** | **[pANGPT1-TEK2] + cbl <-> [pANGPT1-TEK2-cbl]** | **K71=0.5** | **Kr71=0.005** | **(**[**Sasagawa et al, 2005**](#_ENREF_79)**;** [**Yamada et al, 2004**](#_ENREF_102)**)** |
| **r72** | **[pANGPT1-TEK2-cbl] + EPn <-> [pANGPT1-TEK2-cbl-EPn]** | **K72=5** | **Kr72=0.1** | **(**[**Sasagawa et al, 2005**](#_ENREF_79)**;** [**Yamada et al, 2004**](#_ENREF_102)**)** |
| **r73** | **[pANGPT1-TEK2-cbl-EPn] -> cbl + EPn** | **K73=0.001** |  | **(**[**Sasagawa et al, 2005**](#_ENREF_79)**;** [**Yamada et al, 2004**](#_ENREF_102)**)** |
| **r74** | **[pANGPT1-TEK2] + PI3K <-> [pANGPT1-TEK2-PI3K]** | **K74=14** | **Kr74=0.1743** | **(**[**Kiyatkin et al, 2006**](#_ENREF_41)**;** [**Ung et al, 2008**](#_ENREF_92)**)** |
| **r75** | **[pANGPT1-TEK2-PI3K] <-> [pANGPT1-TEK2-pPI3K]** | **K75=33.72** | **Kr75=0.000337** | **(**[**Kiyatkin et al, 2006**](#_ENREF_41)**;** [**Ung et al, 2008**](#_ENREF_92)**)** |
| **r76** | **[pANGPT1-TEK2] + IRS1 <-> [pANGPT1-TEK2 - IRS1]** | **K76=10** | **Kr76=3** | **(Sedaghat et al, 2002)** |
| **r77** | **[pANGPT1-TEK2 - IRS1] -> [pANGPT1-TEK2] + pIRS1** | **K77=0.076** |  | **(Sedaghat et al, 2002)** |
| **r78** | **pIRS1 -> IRS1** | **Kr78=0.023** |  | **(Sedaghat et al, 2002)** |
| **r79** | **TP4 + pPI3K <-> [TP4-pPI3K]** | **K79=1** | **Kr79=0.038** | **(**[**Kiyatkin et al, 2006**](#_ENREF_41)**)** |
| **r80** | **[TP4-pPI3K] -> [TP4-PI3K]** | **K80=0.595** |  | **(**[**Kiyatkin et al, 2006**](#_ENREF_41)**)** |
| **r81** | **[TP4-PI3K] <-> TP4 + PI3K** | **K81=4.7E-06** | **Kr81=2.3E-06** | **(**[**Kiyatkin et al, 2006**](#_ENREF_41)**)** |
| **r82** | **pPI3K + PIP2 <-> [pPI3K-PIP2]** | **K82=25** | **Kr82=3.5** | **(**[**Kiyatkin et al, 2006**](#_ENREF_41)**)** |
| **r83** | **[pPI3K-PIP2] -> pPI3K + PIP3** | **K83=25** |  | **(**[**Kiyatkin et al, 2006**](#_ENREF_41)**)** |
| **r84** | **Akt + PIP3 <-> Akta** | **K84=10** | **Kr84=3** | **(**[**Kiyatkin et al, 2006**](#_ENREF_41)**)** |
| **r85** | **Akta + PDK1 <-> [Akta-PDK1]** | **K85=10** | **Kr85=1** | **(**[**Kiyatkin et al, 2006**](#_ENREF_41)**)** |
| **r86** | **[Akta-PDK1] -> [pAkta-PDK1]** | **K86=10** |  | **(**[**Kiyatkin et al, 2006**](#_ENREF_41)**)** |
| **r87** | **[pAkta-PDK1] <-> pAkta + PDK1** | **K87=0.1** | **Kr87=0.005** | **(**[**Kiyatkin et al, 2006**](#_ENREF_41)**)** |
| **r88** | **pAkta <-> pAkt + PIP3** | **K88=1** | **Kr88=0.001** | **(**[**Kiyatkin et al, 2006**](#_ENREF_41)**)** |
| **r89** | **[pAkt-Takt] -> [Akt-Takt]** | **K89=0.05** |  | **(**[**Kiyatkin et al, 2006**](#_ENREF_41)**)** |
| **r90** | **[Akt-Takt] <-> Akt + Takt** | **K90=0.001** | **Kr90=0.001** | **(**[**Kiyatkin et al, 2006**](#_ENREF_41)**)** |
| **r91** | **pAkt + Takt <-> [pAkt-Takt]** | **K91=10** | **Kr91=1** | **(**[**Kiyatkin et al, 2006**](#_ENREF_41)**)** |
| **r92** | **pAkta + Takt <-> [pAkta-Takt]** | **K92=10** | **Kr92=1** | **(**[**Kiyatkin et al, 2006**](#_ENREF_41)**)** |
| **r93** | **[pAkta-Takt] -> [Akta-Takt]** | **K93=0.05** |  | **(**[**Kiyatkin et al, 2006**](#_ENREF_41)**)** |
| **r94** | **[Akta-Takt] <-> Akta + Takt** | **K94=0.001** | **Kr94=0.001** | **(**[**Kiyatkin et al, 2006**](#_ENREF_41)**)** |
| **r95** | **[pAkta-PDK1] + Takt <-> [pAkta-PDK1-Takt]** | **K95=10** | **Kr95=1** | **(**[**Kiyatkin et al, 2006**](#_ENREF_41)**)** |
| **r96** | **[pAkta-PDK1-Takt] -> [Akta-PDK1-Takt]** | **K96=0.05** |  | **(**[**Kiyatkin et al, 2006**](#_ENREF_41)**)** |
| **r97** | **[Akta-PDK1-Takt] <-> [Akta-PDK1] + Takt** | **K97=0.001** | **Kr97=0.001** | **(**[**Kiyatkin et al, 2006**](#_ENREF_41)**)** |
| **r98** | **Raf1active + pAkt_total -> pRaf1active + pAkt_total** | **K98= 0.1** | **K98= 0.2** | **(**[**Kiyatkin et al, 2006**](#_ENREF_41)**)** |
| **r99** | **pRaf1active -> Raf1active** | **K99=1** |  | **(**[**Kiyatkin et al, 2006**](#_ENREF_41)**)** |
| **r100** | **pAkta + CREB <-> [pAkta-CREB]** | **K100=1.2** | **Kr100=3** | **Estimation** |
| **r101** | **[pAkta-CREB] -> pAkta + pCREB** | **K101=2.7** |  | **Estimation** |
| **r102** | **pCREB + Mcl1 <-> [pCREB-Mcl1]** | **K102=10** | **Kr102=3** | **Estimation** |
| **r103** | **[pCREB-Mcl1] -> pCREB + pMcl1** | **K103=1** |  | **Estimation** |
| **r104** | **pCREB -> CREB** | **K104=1** |  | **Estimation** |
| **r105** | **pMcl1 -> Mcl1** | **K105=1.5** |  | **Estimation** |
| **r106** | **pAkta + mTORC1 <-> [pAkta-mTORC1]** | **K106=1.2** | **Kr106=3** | **(Dalle et al, 2014; Sonntag et al, 2012)** |
| **r107** | **[pAkta-mTORC1] -> pAkta + pmTORC1** | **K107=2.7** |  | **(Dalle et al, 2014; Sonntag et al, 2012)** |
| **r108** | **pmTORC1 + EIFEBP1 <-> [pmTORC1-EIFEBP1]** | **K108=10** | **Kr108=3** | **(Appuhamy et al, 2011)** |
| **r109** | **[pmTORC1-EIFEBP1] <-> pmTORC1 + pEIFEBP1** | **K109=1.54** |  | **(Appuhamy et al, 2011)** |
| **r110** | **pmTORC1 -> mTORC1** | **K110=3.18** |  | **(Dalle et al, 2014; Sonntag et al, 2012)** |
| **r111** | **pEIFEBP1 -> EIFEBP1** | **Kr111=1.02** |  | **(Appuhamy et al, 2011)** |
| **r112** | **PIP3 + PIP3 -> PIP2 + PIP3** | **K112=17** |  | **(Yamada et al, 2003)** |

**2- rules**

| **rule1** | **pAkt_total = pAkt + pAkta** | **Repeated Assignment** |
| --- | --- | --- |

**Table S2**

**1- Nonzero species for SF:**

| **Name** | **Concentration**  **(µM)** | **References** |
| --- | --- | --- |
| **ANGPT1** | **0.00897** | **(Engin et al., 2012)** |
| **TEK** | **0.013492** | **(Engin et al., 2012)** |
| **Shc** | **1** | **(**[**Sasagawa et al, 2005**](#_ENREF_79)**)** |
| **Grb2** | **1** | **(**[**Sasagawa et al, 2005**](#_ENREF_79)**)** |
| **SOS** | **0.3** | **(**[**Sasagawa et al, 2005**](#_ENREF_79)**)** |
| **Ras-GDP** | **0.15** | **(**[**Sasagawa et al, 2005**](#_ENREF_79)**)** |
| **Ras-GAP** | **0.1** | **(**[**Sasagawa et al, 2005**](#_ENREF_79)**)** |
| **Raf1** | **0.5** | **(**[**Sasagawa et al, 2005**](#_ENREF_79)**)** |
| **SHP2** | **0.1** | **(**[**Sasagawa et al, 2005**](#_ENREF_79)**)** |
| **MEK** | **0.68** | **(**[**Sasagawa et al, 2005**](#_ENREF_79)**)** |
| **ERK** | **0.4** | **(**[**Sasagawa et al, 2005**](#_ENREF_79)**)** |
| **MYC** | **0.5** | **(Sears et al, 2000; Lepique et al, 2004; Salghetti et al, 1999; Kim et al, 2003)** |
| **Pase** | **0.5** | **(**[**Sasagawa et al, 2005**](#_ENREF_79)**)** |
| **Pase2** | **0.02** | **(**[**Sasagawa et al, 2005**](#_ENREF_79)**)** |
| **Pase3** | **0.002** | **(**[**Sasagawa et al, 2005**](#_ENREF_79)**)** |
| **IRS1** | **0.076** | **(Sedaghat et al, 2002)** |
| **PI3K** | **0.2** | **(**[**Kiyatkin et al, 2006**](#_ENREF_41)**)** |
| **PIP2** | **0.5** | **(**[**Kiyatkin et al, 2006**](#_ENREF_41)**)** |
| **Akt** | **0.1** | **(**[**Kiyatkin et al, 2006**](#_ENREF_41)**)** |
| **PDK1** | **0.1** | **(**[**Kiyatkin et al, 2006**](#_ENREF_41)**)** |
| **cbl** | **0.8** | **(**[**Sasagawa et al, 2005**](#_ENREF_79)**)** |
| **EPn** | **0.5** | **(**[**Sasagawa et al, 2005**](#_ENREF_79)**)** |
| **Takt** | **0.1** | **(**[**Kiyatkin et al, 2006**](#_ENREF_41)**)** |
| **TP4** | **0.2** | **(**[**Kiyatkin et al, 2006**](#_ENREF_41)**)** |
| **CREB** | **0.2** | **Estimation** |
| **Mcl1** | **0.2** | **Estimation** |
| **mTORC1** | **0.1** | **(Dalle et al, 2014; Sonntag et al, 2012)** |
| **EIF4EBP1** | **0.1** | **(Appuhamy et al, 2011)** |

**2- Nonzero species for DF:**

| **Name** | **Concentration**  **(µM)** | **References** |
| --- | --- | --- |
| **ANGPT1** | **0.01794** | **Estimated** |
| **TEK** | **0.03373** | **Estimated** |
| **Shc** | **1** | **(**[**Sasagawa et al, 2005**](#_ENREF_79)**)** |
| **Grb2** | **1** | **(**[**Sasagawa et al, 2005**](#_ENREF_79)**)** |
| **SOS** | **0.3** | **(**[**Sasagawa et al, 2005**](#_ENREF_79)**)** |
| **Ras-GDP** | **0.15** | **(**[**Sasagawa et al, 2005**](#_ENREF_79)**)** |
| **Ras-GAP** | **0.1** | **(**[**Sasagawa et al, 2005**](#_ENREF_79)**)** |
| **Raf1** | **0.5** | **(**[**Sasagawa et al, 2005**](#_ENREF_79)**)** |
| **SHP2** | **0.1** | **(**[**Sasagawa et al, 2005**](#_ENREF_79)**)** |
| **MEK** | **0.68** | **(**[**Sasagawa et al, 2005**](#_ENREF_79)**)** |
| **ERK** | **0.4** | **(**[**Sasagawa et al, 2005**](#_ENREF_79)**)** |
| **MYC** | **0.025** | **Estimated** |
| **Pase** | **0.5** | **(**[**Sasagawa et al, 2005**](#_ENREF_79)**)** |
| **Pase2** | **0.02** | **(**[**Sasagawa et al, 2005**](#_ENREF_79)**)** |
| **Pase3** | **0.002** | **(**[**Sasagawa et al, 2005**](#_ENREF_79)**)** |
| **IRS1** | **0.076** | **(Sedaghat et al, 2002)** |
| **PI3K** | **0.2** | **(**[**Kiyatkin et al, 2006**](#_ENREF_41)**)** |
| **PIP2** | **0.5** | **(**[**Kiyatkin et al, 2006**](#_ENREF_41)**)** |
| **Akt** | **0.1** | **(**[**Kiyatkin et al, 2006**](#_ENREF_41)**)** |
| **PDK1** | **0.1** | **(**[**Kiyatkin et al, 2006**](#_ENREF_41)**)** |
| **cbl** | **0.8** | **(**[**Sasagawa et al, 2005**](#_ENREF_79)**)** |
| **EPn** | **0.5** | **(**[**Sasagawa et al, 2005**](#_ENREF_79)**)** |
| **Takt** | **0.1** | **(**[**Kiyatkin et al, 2006**](#_ENREF_41)**)** |
| **TP4** | **0.2** | **(**[**Kiyatkin et al, 2006**](#_ENREF_41)**)** |
| **CREB** | **0.2** | **Estimation** |
| **Mcl1** | **0.4** | **Estimation** |
| **mTORC1** | **0.1** | **(Dalle et al, 2014; Sonntag et al, 2012)** |
| **EIF4EBP1** | **0.3** | **Estimation** |

1. **Nonzero species for DF+miRNAs:**

| **Name** | **Concentration**  **(µM)** | **References** |
| --- | --- | --- |
| **ANGPT1** | **0.01794** | **Estimation** |
| **TEK** | **0.2** | **Estimation** |
| **Shc** | **1** | **(**[**Sasagawa et al, 2005**](#_ENREF_79)**)** |
| **Grb2** | **1** | **(**[**Sasagawa et al, 2005**](#_ENREF_79)**)** |
| **SOS** | **0.3** | **(**[**Sasagawa et al, 2005**](#_ENREF_79)**)** |
| **Ras-GDP** | **0.15** | **(**[**Sasagawa et al, 2005**](#_ENREF_79)**)** |
| **Ras-GAP** | **0.1** | **(**[**Sasagawa et al, 2005**](#_ENREF_79)**)** |
| **Raf1** | **0.5** | **(**[**Sasagawa et al, 2005**](#_ENREF_79)**)** |
| **SHP2** | **0.1** | **(**[**Sasagawa et al, 2005**](#_ENREF_79)**)** |
| **MEK** | **0.68** | **(**[**Sasagawa et al, 2005**](#_ENREF_79)**)** |
| **ERK** | **0.4** | **(**[**Sasagawa et al, 2005**](#_ENREF_79)**)** |
| **MYC** | **0.02** | **Estimated** |
| **Pase** | **0.5** | **(**[**Sasagawa et al, 2005**](#_ENREF_79)**)** |
| **Pase2** | **0.02** | **(**[**Sasagawa et al, 2005**](#_ENREF_79)**)** |
| **Pase3** | **0.002** | **(**[**Sasagawa et al, 2005**](#_ENREF_79)**)** |
| **IRS1** | **0.076** | **(Sedaghat et al, 2002)** |
| **PI3K** | **0.2** | **(**[**Kiyatkin et al, 2006**](#_ENREF_41)**)** |
| **PIP2** | **0.5** | **(**[**Kiyatkin et al, 2006**](#_ENREF_41)**)** |
| **Akt** | **0.1** | **(**[**Kiyatkin et al, 2006**](#_ENREF_41)**)** |
| **PDK1** | **0.1** | **(**[**Kiyatkin et al, 2006**](#_ENREF_41)**)** |
| **cbl** | **0.8** | **(**[**Sasagawa et al, 2005**](#_ENREF_79)**)** |
| **EPn** | **0.5** | **(**[**Sasagawa et al, 2005**](#_ENREF_79)**)** |
| **Takt** | **0.1** | **(**[**Kiyatkin et al, 2006**](#_ENREF_41)**)** |
| **TP4** | **0.2** | **(**[**Kiyatkin et al, 2006**](#_ENREF_41)**)** |
| **CREB** | **0.2** | **Estimation** |
| **Mcl1** | **0.4** | **Estimation** |
| **mTORC1** | **0.1** | **(Dalle et al, 2014; Sonntag et al, 2012)** |
| **EIF4EBP1** | **0.3** | **Estimation** |
| **miRNAs** | **1.0E-4** | **(Rabinowits et , 2009; Taylor et al, 2008)** |

**Appuhamy JADRN, Hanigan MD. 2011. Modeling the effects of insulin and amino acids on the phosphorylation of mTOR, Akt, and 4EBP1 in mammary cells. In book: Modelling nutrient digestion and utilisation in farm animals, pp.225-232.**

**Dalle Pezze P, Nelson G, Otten EG, Korolchuk VI, Kirkwood TB, von Zglinicki T, Shanley DP. Dynamic modelling of pathways to cellular senescence reveals strategies for targeted interventions. PLoS Comput. Biol. 2014 Aug; 10(8): e1003728.**

**Engin H, Üstünda Y, Tekin IO, Gökmen A, S¸ehmuz E, ˙Ilikhan SU. 2012. Plasma concentrations of angiopoietin-1, angiopoietin-2 and Tie-2 in colon cancer. Eur. Cytokine Netw. 23 : 68-71.**

**Hsieh, M.Y., et al., Spatio-temporal modeling of signaling protein recruitment to EGFR. BMC Syst Biol, 2010. 4: p. 57.**

**Bidkhori G, Moeini A, Masoudi-Nejad A (2012) Modeling of Tumor Progression in NSCLC and Intrinsic Resistance to TKI in Loss of PTEN Expression. PLoS ONE 7(10): e48004. doi:10.1371/journal.pone.0048004**

**Kholodenko, B.N., et al., Quantification of short term signaling by the epidermal growth factor receptor. J Biol Chem, 1999. 274(42): p. 30169-81.**

**Kim SY, Herbst A, Tworkowski KA, Salghetti SE, Tansey WP (2003) Skp2 regulates Myc protein stability and activity. Molecular Cell 11: 1177-1188.**

**Kiyatkin, A., et al., Scaffolding protein Grb2-associated binder 1 sustains epidermal growth factor-induced mitogenic and survival signaling by multiple positive feedback loops. J Biol Chem, 2006. 281(29): p. 19925-38.**

**Lepique AP, Moraes MS, Rocha KM, Eichler CB, Hajj GNM, et al. (2004) c-Myc protein is stabilized by fibroblast growth factor 2 and destabilized by ACTH to control cell cycle in mouse Y1 adrenocortical cells. J Mol Endocrinol 33: 623-638.**

**Rabinowits G., Gerçel-Taylor C., Day J.D., Taylor D.D., Kloecker G.H. 2009. Exosomal MicroRNA: A Diagnostic Marker for Lung Cancer. Clinical Lung Cancer, Vol. 10, No. 1, 42-46.**

**Salghetti SE, Kim SY, Tansey WP (1999) Destruction of Myc by ubiquitin-mediated proteolysis: cancer-associated and transforming mutations stabilize Myc. Embo Journal 18: 717-726.**

**Sasagawa, S., et al., Prediction and validation of the distinct dynamics of transient and sustained ERK activation. Nat Cell Biol, 2005. 7(4): p. 365-73.**

**Sears R, Nuckolls F, Haura E, Taya Y, Tamai K, et al. (2000) Multiple Ras-dependent phosphorylation pathways regulate Myc protein stability. Genes & Development 14: 2501-2514.**

**Sedaghat, A.R.,Sherman,A.,Quon,M.J.,2002.Amathematicalmodelofmetabolic insulin signaling pathways. Am.J.Physiol.Endocrinol.Metab.283, E1084–E1101.**

**Schoeberl, B., et al., Computational modeling of the dynamics of the MAP kinase cascade activated by surface and internalized EGF receptors. Nat Biotechnol, 2002. 20(4): p. 370-5.**

**Sonntag AG, Dalle Pezze P, Shanley DP, Thedieck K. A modelling-experimental approach reveals insulin receptor substrate (IRS)-dependent regulation of adenosine monosphosphate-dependent kinase (AMPK) by insulin. FEBS J. 2012 Sep; 279(18): 3314-3328.**

**Taylor D.D., Taylor C.G. 2008. MicroRNA signatures of tumor-derived exosomes as diagnostic biomarkers of ovarian cancer. Gynecologic Oncology 110 ;13–21.**

**Ung, C.Y., et al., Simulation of the regulation of EGFR endocytosis and EGFR-ERK signaling by endophilin-mediated RhoA-EGFR crosstalk. FEBS Lett, 2008. 582(15): p. 2283-90.**

**Yamada, S., T. Taketomi, and A. Yoshimura, Model analysis of difference between EGF pathway and FGF pathway. Biochem Biophys Res Commun, 2004. 314(4): p. 1113-20.**

**Yamada, S., et al., Control mechanism of JAK/STAT signal transduction pathway. FEBS Lett, 2003. 534(1-3): p. 190-6.**

**Table S3**

**Dominant follicles in the presence of miRNAs (DF+ miRNAs) model:**

1. **Modified reactions**

| **reactions** | | **parameters** | | **References** |
| --- | --- | --- | --- | --- |
| **r113** | **miRNA + MYC <-> [miRNA-MYC]** | **K113=0.2** | **Kr1130.02** | **(Morozova et al, 2012)** |
| **r114** | **[miRNA-MYC] -> dMYC + miRNA** | **K114=0.1** |  | **(Morozova et al, 2012)** |
| **r115** | **miRNA + TEK <-> [miRNA-TEK]** | **K115=0.2** | **Kr115=0.02** | **(Morozova et al, 2012)** |
| **r116** | **[miRNA-TEK] -> TEK + dmiRNA** | **K116=0.1** |  | **(Morozova et al, 2012)** |

**Morozova N., Zinovyev A., Nonne N., Pritchard L.L., Gorban A.N., Harel-Bellan A. 2012. Kinetic signatures of microRNA modes of action. RNA (2012), 18:1635–1655.**
